# Electrographic flow mapping for atrial fibrillation: theoretical basis and preliminary observations

**DOI:** 10.1007/s10840-022-01308-8

**Published:** 2022-08-15

**Authors:** David E. Haines, Melissa H. Kong, Peter Ruppersberg, Philip Haeusser, Boaz Avitall, Tamas Szili-Torok, Atul Verma

**Affiliations:** 1 Department of Cardiovascular Medicine, Beaumont Hospital, Oakland University William Beaumont School of Medicine, 3601 West 13 Mile Rd., Royal Oaks, MI 48973 USA; 2 Ablacon Inc., Wheat Ridge, CO USA; 3 Department of Medicine and Bioengineering, University of Illinois, Chicago, IL USA; 4 Department of Cardiology, Electrophysiology, Erasmus Medical Center, University Medical Center Rotterdam, Rotterdam, The Netherlands; 5 Southlake Regional Health Centre, Division of Cardiology, University of Toronto, 602-581 Davis Drive, Newmarket, Ontario L3Y 2P6 Canada

**Keywords:** Electrographic flow mapping, Atrial fibrillation, Catheter ablation, Signal processing

## Abstract

Ablation strategies remain poorly defined for persistent atrial fibrillation (AF) patients with recurrence despite intact pulmonary vein isolation (PVI). As the ability to perform durable PVI improves, the need for advanced mapping to identify extra-PV sources of AF becomes increasingly evident. Multiple mapping technologies attempt to localize these self-sustained triggers and/or drivers responsible for initiating and/or maintaining AF; however, current approaches suffer from technical limitations. Electrographic flow (EGF) mapping is a novel mapping method based on well-established principles of optical flow and fluid dynamics. It enables the full spatiotemporal reconstruction of organized wavefront propagation within the otherwise chaotic and disorganized electrical conduction of AF. Given the novelty of EGF mapping and relative unfamiliarity of most clinical electrophysiologists with the mathematical principles powering the EGF algorithm, this paper provides an in-depth explanation of the technical/mathematical foundations of EGF mapping and demonstrates clinical applications of EGF mapping data and analyses.

**Graphical abstract:**

Starting with a 64-electrode basket catheter, unipolar EGMs are recorded and processed using an algorithm to visualize the electrographic flow and highlight the location of high prevalence AF “source” activity. The AF sources are agnostic to the specific mechanisms of source signal generation.

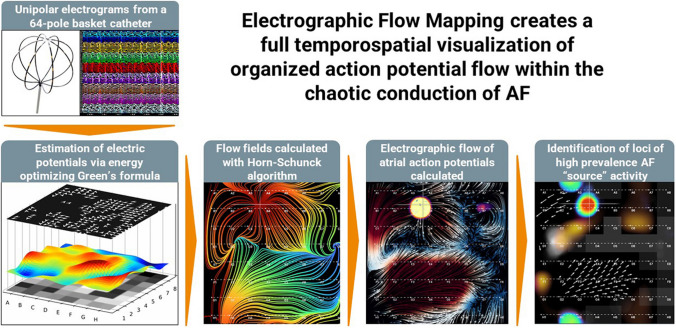

## Introduction

Catheter ablation to electrically isolate the pulmonary veins (PVs) provides the cornerstone for treatment of paroxysmal atrial fibrillation (AF); however, the effectiveness of catheter ablation for the treatment of persistent and long-standing persistent AF remains below expectations. Outcomes associated with current techniques indicate that there are opportunities for improvement and innovation [1, 2]. Consensus does not exist regarding the optimal ablation strategy for patients with persistent AF, and this has resulted in a proliferation of methods for identifying clinically relevant adjunctive, extra-PV ablation targets. Increasing evidence from both preclinical and clinical studies support the role of self-sustained, non-PV, spatially localized triggers or drivers that initiate and maintain AF [3–5]. Several algorithm-based approaches for localizing non-PV AF triggers and drivers have been developed but have resulted in inconsistent clinical outcomes [6–11]. Currently available mapping technologies suffer from many technical limitations including variable signal quality, high sensitivity to noise and artifacts, susceptibility to timing errors and morphology correlation, inability to demonstrate time-dependent electrical behaviors and real-time or beat-by-beat mapping [12].

Electrographic flow (EGF) mapping is an innovative approach to cardiac mapping that does not rely upon phase analysis or other traditional signal analysis methods. Rather, EGF employs the same principles used for optical flow and fluid dynamics to estimate cardiac electrographic “flow” traveling through the atrial myocardium because of transmembrane voltage changes represented by unipolar electrograms (EGMs) [13, 14]. Low density basket electrode catheters allow the operator to map large segments of a cardiac chamber with high temporal resolution. However, because of spatial resolution limitations inherent in mapping with this type of catheter, EGF mapping may be used to identify regions of coherent atrial electrical activation with normal or near normal conduction velocities rather than trying to decipher complex conduction patterns in regions of slow conduction and block. EGF mapping allows for the full spatiotemporal reconstruction of organized electrographic activity within the otherwise chaotic and disorganized electrical conduction of AF and thereby identifies source regions from which EGF flow originate and propagate in a centrifugal pattern. It allows for the differentiation between active sources of electrical activation (centrifugal activation) and passive rotational phenomena (centripetal activation) [13]. The purpose of this white paper is threefold: (1) to provide an in-depth explanation of the technical and mathematical foundations of EGF mapping algorithms; (2) to describe the pre-processing pipeline and mapping algorithm; and (3) to demonstrate the clinical applications of the EGF mapping data and analyses.

## Baseline assumptions and definitions

As debate still exists around the precise underlying pathophysiologic mechanisms of AF, we must start by outlining the fundamental tenets and definitions required for EGF mapping:

1. AF is triggered or driven by sources of excitation which generate excitation wavefronts [15].
2. The mechanisms of localized source activation may be varied, including automaticity, triggered activity [16], functional reentry including rotors [17], anatomically determined micro-reentry [5], and epicardial to endocardial breakthrough from longitudinal dissociation of endocardial and epicardial activation wavefronts [18].
3. Such sources are often located in the PVs but may occur at any location in the atrial tissue [19].
4. Sources present themselves as origins of divergent excitation waves and can be detected by tracking the excitation wave propagation in the myocardium around them.

Atrial sources that produce excitatory waves during ongoing AF may be detectable for varying durations of time. Those sources that are more durable and entrain a wider atrial area may increase the risk of AF recurrence despite successful PV isolation, and thus may be relevant ablation targets.

## Panoramic near-field EGM recording

**Fig. 1:**
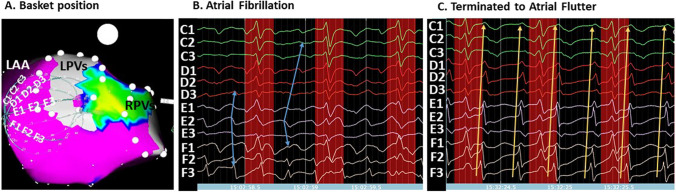
Panoramic near-field electrogram recording with a basket catheter. **A** A 50 mm basket catheter (FIRMap™, Abbott, Abbott Park, IL) is shown in a left lateral position within the left atrium (LA) of a persistent atrial fibrillation (AF) patient. **B** During AF, unipolar electrograms (EGMs) show an irregular and variably-correlated pattern with earliest activation at E2 (blue arrows). Ablation at electrodes GH1 resulted in AF termination to an atypical LA flutter. **C** Unipolar EGMs obtained during atypical atrial flutter now demonstrate highly correlated and regular activation pattern with F3 as the point of earliest activation (orange arrows)

Using multi-electrode basket catheters for panoramic near-field EGM recording has previously been reported by several groups. However, there are many limitations to interpreting activation sequences through the basket information alone [10, 20, 21]. Correctly placed, basket catheters can touch the endocardial surface of either the right (RA) or left atrium (LA) with a large number of their electrodes which enables the recording of unipolar EGMs in a grid with electrodes spaced 1 to 2 cm apart (Fig. 1). In this example, a single ablation at electrodes G1/H1 terminated AF into an atypical LA flutter (Fig. 1C). During flutter, the EGMs became completely regular and highly correlated with a uniformly laminar electrographic flow. Estimating delays between activation times by direct correlation of neighboring, well-correlated electrodes generally yield values below 30 ms in both AF and atrial flutter corresponding to a velocity of the EGF of 66 cm/s which corresponds to a physiologically realistic wavelength of around 10 cm. In less correlated electrodes with highly fractionated potentials (e.g., D2 and D3 in Fig. 1B), such values are much more difficult to define, demonstrating the variability and ambiguity of local activation patterns during AF. Therefore, there is not yet an established way to map activation in AF with low density mapping in the standard time domain.

## Electrographic flow algorithm

To visualize time-dependent activations during AF, we developed a combination of Green’s formula-based spline interpolation with Horn-Schunck’s iterative flow estimation. This combined algorithm requires pure atrial near-field unipolar EGMs, which are normalized as input data (normalized, because multiple factors affect local electrogram amplitudes which are unimportant for this analysis). Hence, raw EGMs are preprocessed according to the sequence outlined in Table 1. Interelectrode interpolation using Green’s formula cannot exclude that signals in between the electrodes may look different, but because excitation waves showing divergent behavior (indicating conduction away from AF sources) have a wavelength of several centimeters, they can be mapped despite low electrode density. The Horn-Schunck algorithm is designed to interpret the movements of objects in video sequences [22], but is even better in interpreting excitation waves because the brightness constancy constraint of the algorithm is always fulfilled by all-or-none excitation waves.

**Table 1:** Preprocessing of unipolar EGMs from basket recordings

	Step	Description
1	Removal of the QRS complex	Cut out 100–180 ms of data around the R-peak
2	Removal of baseline fluctuations	High pass filter at 5 Hz
3	Removal of common noise and remaining far field signals	All-channel average subtraction
4	Conversion of voltage values of different channels to intensity of equal amplitude	Normalization to identical min–max amplitude range over a window of 900 ms
5	Down-sampling in the time domain to discrete intensity frames	Average the intensity data for each 19 ms

**Fig. 2:**
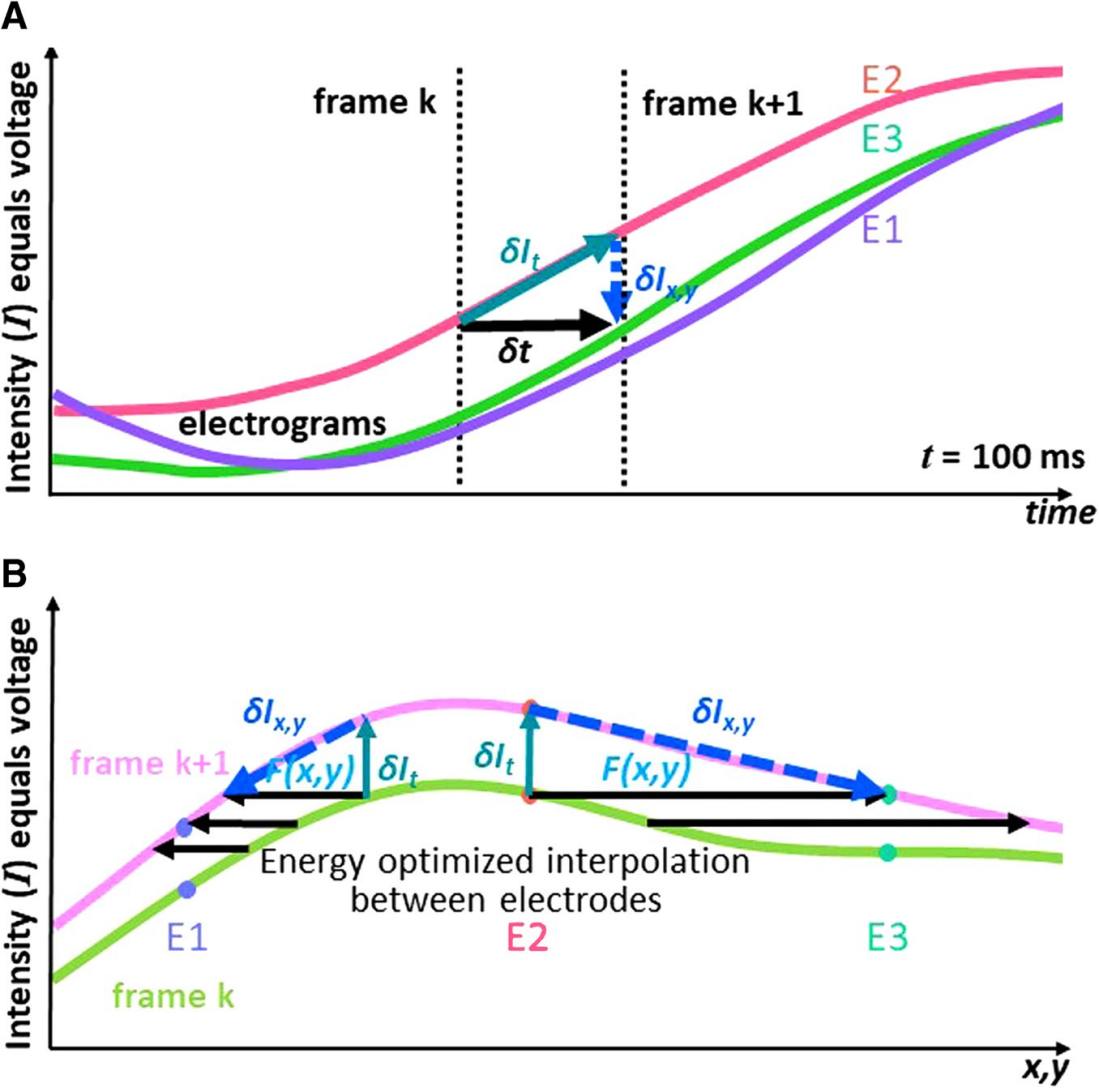
Electrographic flow mapping algorithms convert time domain information from electrograms into space domain of flow vectors. **A** Time-dependent intensities derived from electrodes E1, E2, and E3 from the unipolar electrograms (EGMs) recorded and displayed in Fig. 1D during AF. As the leading electrode, the intensity of E2 rises first (pink trace), while E1 and E3 rise approximately 20 ms later—this represents a potential wave originating at E2 and traveling from there to both sides, E1 and E3. **B** Conversion from the time domain triangle depicted in A to the space domain. See text for details

Figure 2 explains how the two algorithms are combined to convert time domain information from the EGMs into the space domain of flow vectors. The procedure is explained in detail for the intensity data derived from the three electrodes E1, E2, and E3 from the recording in Fig. 1B. Time-dependent intensities derived from these three electrodes are displayed in Fig. 2A. The leading electrode is E2 where intensity rises first (pink trace in Fig. 2A). E3 and E1 show a similar amplitude rise approximately 20 ms later. This corresponds to a potential wave starting at E2 and traveling from there to both sides, E1 and E3. A frame is the image of the mean potential over 19 ms duration. During the time *δt* (the time interval between two consecutive frames k and k + 1), E2 increases in intensity by δ*I*_*t*_*.* δ*I*_*t*_ corresponds to the temporal gradient of the intensity *I*_*t*_ times *δt*. δ*I*_*t*_ in our demonstration example is approximately equal to -*δI*_*x,y*_ (the intensity difference between electrode E2 and E3 at frame k + 1) because the wave front follows the direction of the spline and the wave travels about one electrode distance during *δt*. *δI*_*x,y*_ has been derived from the individual gradients in x and y direction of the Intensity of frame k + 1:

$${\mathrm{\delta I}}_{\mathrm x,\mathrm y}={\mathrm I}_{\mathrm x,\mathrm y}\ast\mathrm{\delta x},\mathrm y=\left(\frac{{\mathrm I}_{\mathrm x}\ast{\mathrm\delta}_{\mathrm x}}{{\mathrm I}_{\mathrm y}\ast{\mathrm\delta}_{\mathrm y}}\right)$$

with ${\delta }_{x}$ and ${\delta }_{y}$ being the spatial distance between E2 and E3. The triangle between the intensity E2 at k and intensity E3 at k + 1 in Fig. 2A is now converted into the space domain in Fig. 2B by applying Green’s formula-based spline interpolation forming an energy optimized smooth 200 × 200 unit $x,y$ intensity surface plain from the original 8 × 8 electrode grid. The vertical axis represents intensity in both graphs while the horizontal axis is time in Fig. 2A but represents distance in Fig. 2B and is a one-dimensional simplified visualization of the two-dimensional atrial surface. On the atrial surface plain, we define the electrographic flow vector ${F}_{x,y}=\left(\frac{u^{k+1}}{v^{k+1}}\right)$ with its components $u^{k+1}$  and $v^{k+1}$ . $u^{k+1}$ represents the x-distance by which the wave travels during *δt* and *v*^*k*+*1*^ represents the y-distance by which the wave travels during *δt.* In the space domain *δI*_*t*_, -*δI*_*x,y*_ and the flow vector (${F}_{x,y}$) also form a triangle which in our demonstration example where the wave front went along spline E with a speed of about one electrode distance per *δt* is approximately matching E2 and E3. In any arbitrary example with unknown wave flow direction, ${F}_{x,y}$ is ill-defined, however, because the intensity surfaces at the frames k and k + 1 are two-dimensional and allow the drawing of multiple possible triangles for multiple combinations of *u*^*k*+*1*^ and *v*^*k*+*1*^. In other words, each pair of frames allows for multiple solutions of possible flow vectors for each matrix point. Moreover, because of the chaotic nature of AF each individual frame pair may yield a different set of possible solutions, repetitive patterns like source activity and rotational phenomena might show reproducible flow patterns which inform the utilization of stacks of multiple frames for analysis. Because of the dynamic nature of AF activation, it seems reasonable on the other hand to use no more than a few seconds of data to determine the momentary status of electrographic flow.

The problem of extracting the consistent flow pattern—i.e., the pattern reproduced in a majority of waves—from tens to hundreds of frames of our intensity matrix is best addressed by the recursive Horn-Schunck algorithm, which had been originally designed to find the best flow vector matrix transforming the intensity pattern of one frame into another of a single frame pair:

$$\mathrm u^{\mathrm k+1}=\overline{\mathrm u}^{\mathrm k}-\frac{{\mathrm I}_{\mathrm x}\left({\mathrm I}_{\mathrm x}\overline{\mathrm u}^{\mathrm k}+{\mathrm I}_{\mathrm y}\overline{\mathrm v}^{\mathrm k}+{\mathrm I}_{\mathrm t}\right)}{4\mathrm\alpha^2+\mathrm I_x^2+\mathrm I_{\mathrm y}^2}$$

$$\mathrm v^{\mathrm k+1}=\overline{\mathrm v}^{\mathrm k}-\frac{{\mathrm I}_{\mathrm y}({\mathrm I}_{\mathrm x}\overline{\mathrm u}^{\mathrm k}+{\mathrm I}_{\mathrm y}\overline{\mathrm v}^{\mathrm k}+{\mathrm I}_{\mathrm t})}{4\alpha^2+\mathrm I_{\mathrm x}^2+\mathrm I_{\mathrm y}^2}$$

In its standard formulation, this algorithm iteratively determines the flow vector X- and Y-components *u*^*k*+*1*^and *v*^*k*+*1*^ from the local average of the previous iteration, ${\overline{u} }^{k}$ and ${\overline{v} }^{k},$ from *I*_t_, and from the spatial intensity gradient *I*_x_ and *I*_y_ from one pair of frames. Iterations are repeated until they obtain minimization of the Horn-Schunck error function:

$${\mathrm E}_{\mathrm k+1}=({\mathrm I}_{\mathrm x,\mathrm y}\overline{\mathrm u}^{\mathrm k}+{\mathrm I}_{\mathrm x,\mathrm y}\overline{\mathrm v}^{\mathrm k}+\mathrm{It})$$

which would equal zero if the triangle based on the previous flow vector of iteration k already fits perfectly into the intensity surface of iteration k + 1. If the triangle based on ${\overline{u} }^{k}$ and ${\overline{v} }^{k}$ does not fit, the Horn-Schunck iteration results in an adaptation of *u*^*k*+*1*^ and *v*^*k*+*1*^.

In the multiple frame variant of Horn-Schunck used here for electrographic flow analysis, we aim for solving the same problem—not for a single frame pair but for a series of frames. Instead of only doing a converging number of iterations on an individual frame, we use consecutive frame pairs for consecutive iterations. At the same time, we increase the factor α, which is kept around unity in single frame pair applications to reduce the effect of an individual frame on the result. Convergence in the multi-frame implementation is reached when the resulting flow vector field reaches asymptotically stable vector length with no more systematic growth with each iteration. Best results for reproducibility and outcomes correlations have been achieved if a single pair of frames undergoes 3 to 7 iterations before the algorithm considers the next frame pair. In total between 50 and 100 frames are needed at an $\alpha$ of 100 to reach convergence at a stable flow vector length.

**Fig. 3:**
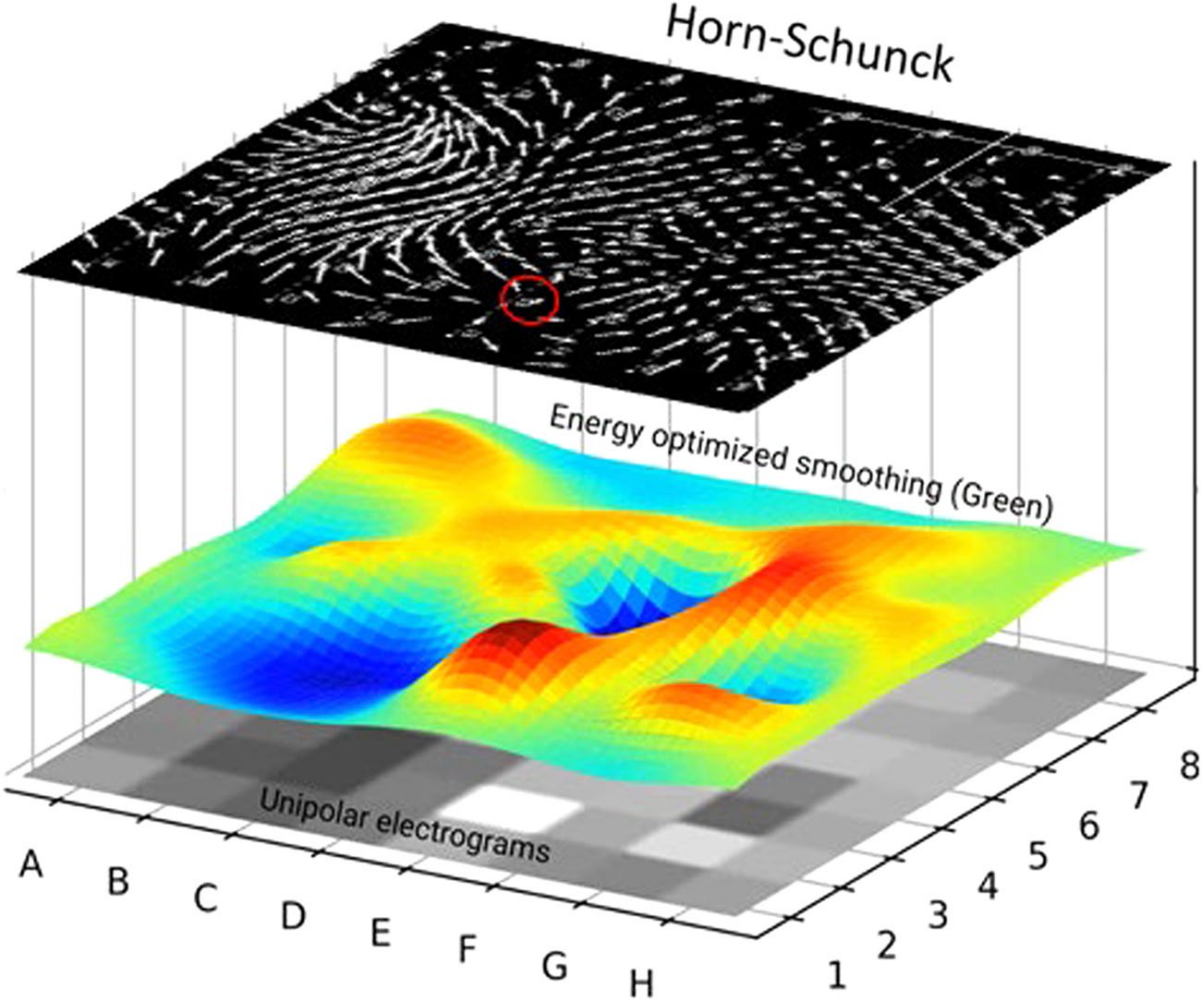
Transformation of intensity values to Horn-Schunck flow field. Illustrated transformation of the intensity values of the 8 × 8 matrix of basket unipolar EGMs (grey squares of lowest layer) into the Green’s function derived energy-optimized intensity surface after the biharmonic spline fit (middle layer) and finally into the arrows of the Horn-Schunck flow field (top layer) after 735 iterations

**Fig. 4:**
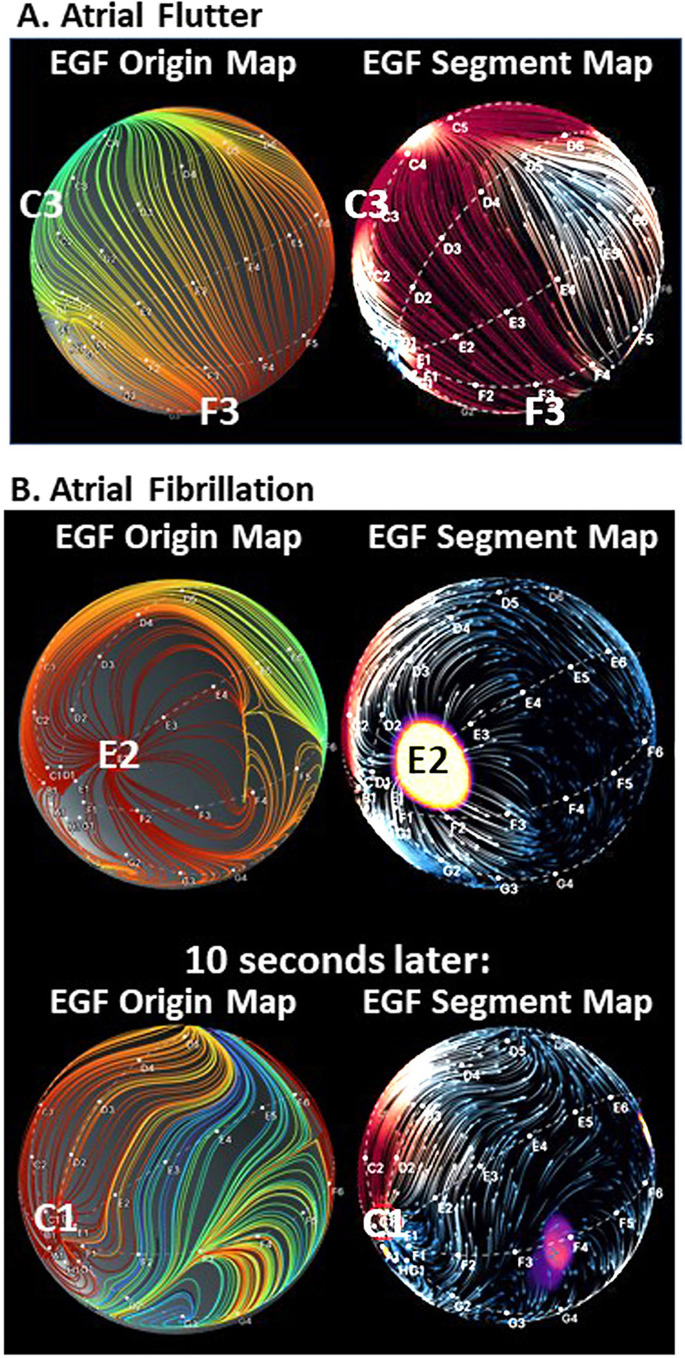
Stability of EGF origin in organized atrial arrhythmia versus atrial fibrillation. **A** 3D EGF Origin Map from 4 s of recording of the atypical atrial flutter shown in Fig. 1C with corresponding EGF Segment Map. **B** 3D EGF Origin Maps with corresponding EGF Segment Maps during AF show that at one point in time (top map pair), the streamlines originate from E2, while 10 s later the flow arises from C1 (bottom map pair), highlighting the time-dependent electrical behavior of AF (see text)

Figure 3 shows an example for the transformation of the intensity values of an 8 × 8 matrix of basket unipolar EGMs (gray squares of the bottom layer) into the Green’s formula interpolated energy-optimized intensity surface in the space domain shown in rainbow color (intermediate layer), and finally, the resulting vector field of the Horn-Schunck flow field (arrows indicate flow distance and direction between two consecutive frames, upper layer) after 735 iterations applied on 105 frames originally recorded for 2 s. Figure 4 shows the EGF result with respect to the recordings from Fig. 1 on a spherical basket model to visually correlate the result with the 3D electroanatomical map images (CARTO, Biosense Webster, Diamond Bar, CA, USA) using visualization of the flow through the movement vectors of imaginary waves. Trace arrows of imaginary wave movement are higher resolution and different from straight flow field vectors represented by EGF segment maps (Fig. 4 right panels), or by mathematical streamlines represented by EGF origin maps (Fig. 4 left panels) which indicate the full path of an imaginary flowing particle and are colored red to blue from the starting point of EGF to its end both defined by zero vectors (singularities). In atrial flutter (Fig. 4A), the flow goes homogenously from F3 to C3 confirming the directionality of flow as already concluded from the activation sequence of the unipolar EGMs. This pattern of EGF origin was found to be very stable for all time segments. In contrast, for the identical catheter position but recording in AF (Fig. 4B) each time segment produced a different result. In the top pair of maps, all streamlines arise from a localized source at electrode E2 while in the second pair of maps recorded 10 s later, the EGF origin map shows that flow arises from the catheter pole electrodes in line with the observed leading position of C1 in the EGMs.

## Sources, singularities, divergence, and switching sources

Despite the high variability of flow fields and corresponding streamline patterns in AF, origins of EGF (sources) were found to often reappear again and again in the same locations. To integrate this repeated source activity over time, EGF mapping software can create source maps that register the prevalence of sources (flow origins surrounded by a divergent flow pattern) using the frame-by-frame iterations of the Horn-Schunck results after reaching flow field convergence (steady state flow fields). By summing up the data from 105 consecutive frames of data for a 2 s segment after reaching steady state, we yield a prevalence of each source at its specific location for this segment of 0 to 100%. The longer recording time was analyzed, the more consistently the specific sites with repetitive source prevalence appeared.

**Fig. 5:**
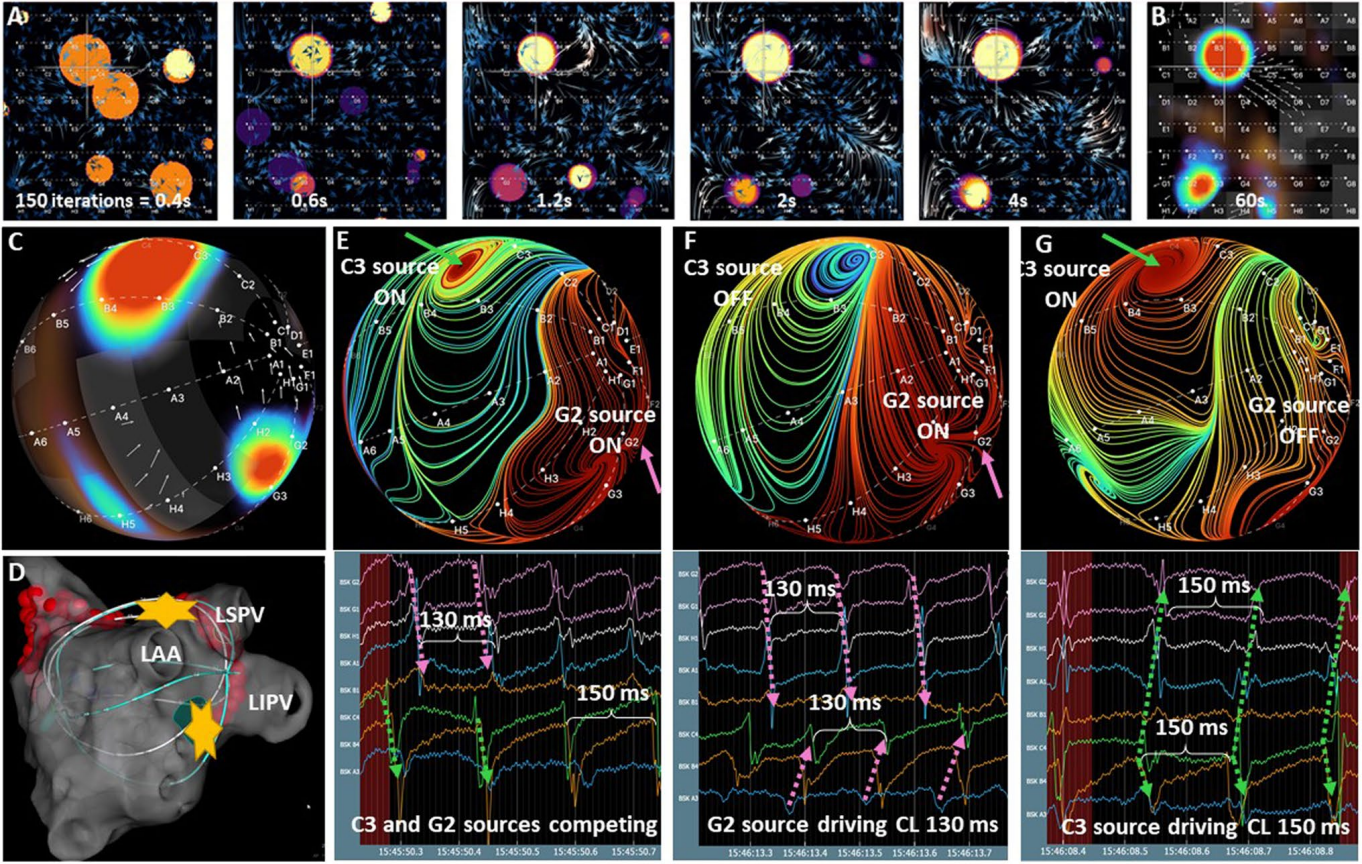
Sources, singularities, divergence, and switching sources. **A** Time-lapse series of frames demonstrates increasing consistency of sources with more iterations. While after 400 ms the flow field remains very undefined and multiple possible sources are estimated, 4 s of Horn-Schunck iterations reveal a pattern of two sources, which is very stable and stayed consistent over the next minute **B** EGF Summary Map obtained the following minute after the 60-s of recording from the series in A, reproducibly shows the same pattern of two stable and consistent sources. **C** 3D version of EGF Summary Map shown in B. **D** Electroanatomic map displaying source at C3 along the ridge between the left atrial appendage (LAA) and left superior pulmonary vein (PV). The second source at G2 is anterior to the left inferior PV. **E** EGF Origin Map generated over 4 s and corresponding unipolar EGMs showing 2 different AF sources with very distinct shapes and fields of influence. Since the activity of both sources was 40% and 33%, respectively, meaning they are only ON for 2/5 and 1/3 of the time, their ON times overlap so that both cycle lengths of 130 and 150 ms can be observed simultaneously in the unipolar EGMs as well as individually depending on which source is active (**E–G**)

In the standard setting, the EGF algorithm analyzes 30 overlapping 4 s segments from a 1-min recording where the first 2 s of each are used to reach steady state and the subsequent 2-s segments are used to determine source intensity. The analysis window duration was selected to optimize between a long enough duration to establish flow patterns but short enough to resolve changing activation patterns. The prevalence values of sources become more and more consistent and reproducible with an increasing number of frames during the training phase in a case where we deal with two primary stable sources of flow (Fig. 5). Duration of acquisition was optimized by comparing source prevalence using varying recording durations in a preliminary series of patients undergoing EGF mapping. It was observed that recording durations of 60 s contributed to map consistency, particularly for less stable sources, but recording durations > 60 s did not meaningfully improve source statistics (unpublished data).

The system records organized as well as disorganized average flow information to identify the location of source activity. Workflow in the clinical procedure lab includes basket electrode placement in the chamber of interest, 1-min signal acquisition, then immediate generation of the flow maps and summation maps. The basket electrode catheter can be repositioned, and maps re-acquired multiple times during the clinical case. The algorithm to generate an EGF map may be applied to any multipolar catheter array with a minimum of 16 recording electrodes, but the greater the electrode number, the greater the mapping coverage of the chamber of interest. Higher electrode density will allow for greater spatial resolution of the anatomical locations of AF sources; however, the system is intentionally designed to display average EGF flows over broad areas of the endocardial surface, thus low density multielectrode catheters can be employed.

By analyzing sufficient data to estimate intensity and location of sources, the resulting source maps are quite spatially and temporally stable. The randomized, controlled FLOW-AF study (NCT 04,473,963) has completed enrollment with expected results to become available upon completion of the 12-month follow-up visits. One of the pre-specified secondary endpoints of the study examines EGF map consistency and reproducibility both intra-procedurally and inter-procedurally. In the interim, inter-procedural reproducibility was reported in a case report that showed high spatiotemporal stability of EGF mapping performed in separate ablation procedures performed 18 months apart [23].

A clinically relevant source activity threshold has been defined based on a retrospective analysis and post-processing of stored unipolar EGM recordings from 64 patients with persistent AF, who underwent FIRM-mapping and ablation [24]. This study correlated source parameters with procedural outcomes at 12 months post-ablation. Patients with active sources (divergent flow emanating from singularity), where a total of > 26% of the 60-s recording duration at the conclusion of whatever ablation procedure was performed were observed, were more likely to have AF recurrence. However, observations of prevalence and number of sources as predictors of ablation outcomes need to be made in larger populations of patients before any prescription based upon EGF maps can be recommended. A preliminary retrospective analysis of 64-pole basket electrode recordings from 405 patients who had undergone attempted AF ablation showed a relatively even distribution of putative AF sources from the right and left atria. The most common sites of origin identified were the SVC/RA junction, the anterior LA, the left atrial appendage and the LAA/LSPV ridge [25].

Source activity was only moderately dependent on spatial distribution of electrodes because EGF-based source activity does not appear to be significantly different between polar locations on the basket vs equatorial locations where electrodes are more distant with a trend to higher values at the polar locations (not shown). Conventional mapping systems may achieve high spatial resolution in small regions and then create an aggregate map if the activation sequence is fixed and repetitive. However, the dynamic pattern of AF conduction precludes this approach. Alternatively, high temporal resolution with lower spatial resolution may be achieved with multielectrode basket catheters but remains inadequate for activation mapping of complex AF circuits. Electrographic flow mapping addresses these limitations. To correlate EGM morphology of AF measured by a high-density regional mapping catheter to the lower density global EGF mapping using a basket mapping catheter, we performed exploratory experiments in a rapid-atrial pacing canine model of self-sustained AF. With the animal in self-sustained, persistent AF of approximately 3 months’ duration, a basket mapping catheter was inserted into the RA and EGF maps were generated from the raw unipolar EGMs. At the locations of active sources and passive rotational phenomena, a high-resolution IntellaNav MiFi™ (Boston Scientific, Natick, MA, USA) catheter with 3 closely spaced mini electrodes was used to interrogate these regions of interest as indicated by the EGF Summary Map. These initial investigations showed that at locations where an active source was identified, there were repetitive salvos of low amplitude, high frequency signals on the closely spaced bipoles of the ablation catheter. Further study is warranted, and a prospective, observational single-center pilot study is currently underway [26].

## Ablation of a stable source

**Fig. 6:**
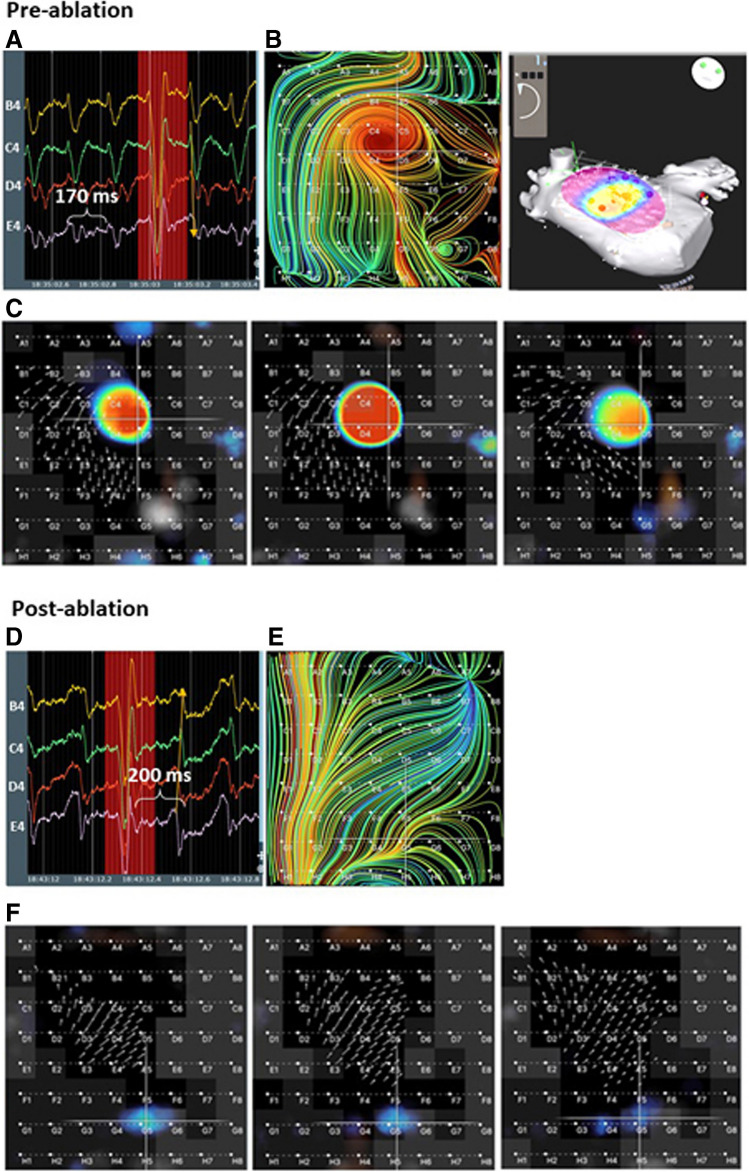
Ablation of a stable source. An EGF-identified active source located in the anterior wall of the LA demonstrates an AF cycle length of 170 ms (**A**) with a rotational flow pattern as shown in the EGF Origin Map (**B**) and a reproducibly stable source activity ranging from 15.1 to 28.9% shown in 3 separate 1-min recordings (**C**). Post-ablation, the AF cycle length has increased to 200 ms (**D**) and the origin of flow (source) is no longer evident in the EGF Origin Map (**E**) nor is source activity detected on any of the 3 separate 1-min EGF Summary Maps (**F**)

Stable sources that generate divergent flow are called “active” and can be focal and rotational in nature. A stable source is practically defined as a location with a prevalence during the mapping acquisition of 5% or greater. A dominant source is defined as a 20% or greater prevalence. If an area of rotation does not generate divergent flow but instead is an area where electrographic flow converges, then it is considered as “passive” rotational pattern. In a prospective analysis by Bellman et al., 25 patients with persistent or long-standing persistent AF, who had previously undergone FIRM-guided ablation underwent processing of their raw unipolar 64-pole basket recordings using the EGF mapping software [13]. In the original FIRM analysis, 43 rotors and 1 focal impulse were identified; however, re-analysis of these same unipolar 64-electrode recordings using EGF mapping software revealed that only 24 of the 43 FIRM rotors were detected as active EGF sources while 16 were passive with centripetal rotational activity and 4 were not visualized at all. Fifteen of these 24 active EGF-confirmed AF sources showed centrifugal rotational patterns and 9 were focal impulses but only 13 had temporal prevalence above 20%. In the same patients, EGF mapping found another 22 sources with > 20% prevalence that were undetected by FIRM. Seventeen were focal, demonstrating that FIRM does not detect the majority of significant focal sources. In the example shown in Fig. 6 a single rotational source (in the anterior wall of the LA of a patient with persistent AF is well reproduced by the EGF map over three separate acquisitions. It was then targeted with RF ablation resulting in its disappearance while the cycle length at the same time increased from 170 to 200 ms. Prevalence of all source activity decreased from an average of 39.6% before ablation to an average of 12.6% after ablation.

## Sources as origins of excitation

**Fig. 7:**
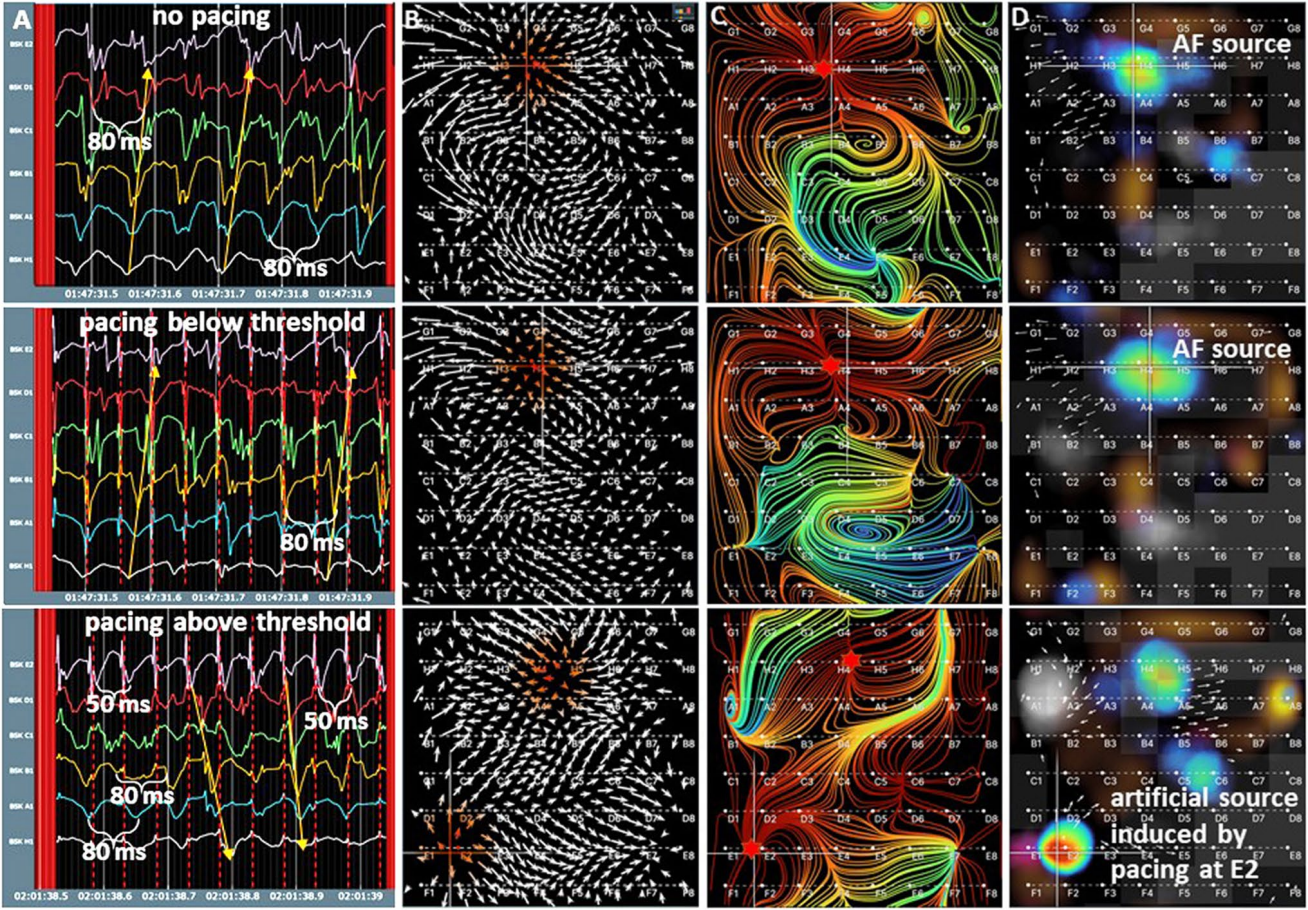
EGF Sources are origins of excitation waves.Top row: Unipolar electrograms (EGMs) recorded during sustained AF in a canine model. Panel **A** shows unipolar EGMs with evidence of a broad activation wavefront. Panel **B** shows the EGF map with a flow field diverging from a putative AF source at electrode H4. Panel **C** presents the EGF Origin Map again demonstrating divergent flow from H4. The EGF Summary Map in panel **D** displays a dominant AF source at site H4 (crosshairs). Middle row: During subthreshold pacing from an ablation catheter at site E2, unipolar EGMs appear unchanged from baseline except for the superimposed pacing artifact. Likewise, there is no substantive change in the flow vector patterns of the overall flow field **(B**), the origins of flow (**C**), or the EGF Summary Map (**D**). Bottom row: Pacing at high output from site E2 reverses the activation sequence of the unipolar EGMs (**A**). Flow vector field now shows a second separate divergent singularity (**B**) and the EGF Origin Map shows flow originating from both the spontaneous AF source location and the location of pacing (**C**). EGF Summary Map also shows both the spontaneous AF source as well as the simulated source induced by pacing at E2 (**D**)

The EGF mapping algorithm to detect sources is designed to recognize divergent electrical activation starting from a singularity of flow vectors. To demonstrate that the origin of excitation can be recognized by these attributes (singularity and divergence), we performed EGF mapping in a canine model of persistent AF [27]. The dominant sources of spontaneous self-sustained AF were mapped first and then focal activation from a remote source was simulated with rapid atrial pacing from an ablation catheter **(**Fig. 7). When subthreshold pacing was initiated, no localized source at that site was identified with EGF mapping, but when higher pacing output resulted in local capture, a localized source with divergent activation appeared on the EGF map in the location of the pacing stimulus.

Other mapping technologies employed in canine models of fibrillatory conduction have not been able to correctly localize pacing locations simulating a focal activation and have instead been shown to frequently identify spurious focal activation sites and epiphenomenal rotational activation sites [28].

## Limitations of existing mapping options

While the mechanisms of AF initiation and maintenance remain incompletely elucidated, there is growing evidence for the presence of self-sustaining extra-PV drivers and/or triggers that maintain AF and these localized AF sources may represent adjunctive ablation targets beyond PVI [19, 29–32]. A proliferation of AF mapping techniques attempt to localize these self-sustained triggers and/or drivers responsible for initiating and/or maintaining AF [7, 8, 11, 33–36]. Nonetheless, current approaches suffer from technical limitations including sensitivity to errors of timing and morphology correlation; susceptibility to signal artifact and noise; and poor spatiotemporal stability as well as the inability to distinguish active from passive electrical phenomena [37].

Both intracardiac EGM-based quantitative approaches as well as other phase and/or activation mapping approaches have been put forth for the identification of relevant extra-PV sources of AF. Nominal signal analysis methods for detecting AF sources by targeting locations with maximal dominant frequency (DF) or with complex fractionated atrial electrograms (CFAEs) have yielded inconsistent clinical results due to the variability in methodologies used, reliance on empirically derived definitions and their inherent technical limitations including sensitivity to EGM signal amplitude and morphology, timing errors, and signal quality and noise [12, 38–43]. Additionally, multiple algorithm-based mapping systems have also been developed to identify extra-PV AF sources using a wide variety of signal recording and processing techniques; however, currently available AF recording and processing technologies remain restricted to specific applications or suffer from technological limitations as summarized in detail in a recent position paper published by the European Heart Rhythm Association and European Society of Cardiology Working Group [44].

## Limitations of EGF mapping

As is found with all algorithms used to simplify complex systems, Green’s formula and the Horn-Schunck algorithm employed in EGF mapping with low density basket electrodes result in limitations in both spatial and temporal resolution. The resolution of source detection is in the range of the electrode distance and would define the size of the area to be ablated upon detection of a relevant source and cannot differentiate between a single source or multiple closely spaced sources. EGF does not discriminate organized from disorganized wavefronts except by flow consistency, which would be lower in the latter case. Flow consistency does not influence source detection or source activity, however. Down-sampling of the unipolar EGMs was performed by averaging the intensity data (voltage) for each 19 ms window, which is effectively a form of low-pass filtering. Interestingly, when shorter intervals between frames were tested, this led to a lower signal-to-noise ratio. The algorithm looks for directionality of mean propagation with a time resolution of 4 s and a sampling rate of 2 s. In other words, EGF detects only changes of the average flow over 4 s and records this value every 2 s.

The primary limitations to EGF mapping relate to the use of a panoramic basket catheter. In addition to spline bunching and/or splaying, the issue of basket non-conformation to most atrial chambers results in non-uniform endocardial contact and incomplete atrial coverage. As such, 2 to 3 basket positions are required in each atrium to adequately cover the endocardial surface. Similarly, non-ideal and non-uniform electrode contact due to basket non-conformation can result in low intracardiac signal-to-noise ratios and the EGF estimation may over-interpolate in regions where multiple electrodes suffer poor wall contact. Insensitivity to mapping details of the rhythm in areas of slowed conduction or in cases where the distance between two sources is less than the interelectrode distance are inherent in the system; however, a possible solution is to combine low-density basket mapping with high-density local mapping. The next version of the EGF mapping software contains a feature that quantifies the near-field component of signals per electrode as an indication of the f-wave signal quality. Also related to the issue of basket conformation is the differential detection of source activity near the poles due to the geometric projection and variable energy density due to unequal spatial sampling. This risk can be mitigated by scaling activity values as they approach the poles of the basket. Another limitation of EGF mapping is that the current version of the software simply removes the QRS complex, which diminishes the signal remaining for analysis particularly at faster ventricular response rates in AF. In addition to imposing an upper heart rate limitation to the signal processing, this could potentially lead to artifact if the algorithm detects a T-wave as an f-wave in situations where the T-wave is particularly prominent, such as when the basket it positioned near a valve. The next version of the software now uses a QRST subtraction algorithm.

## Future directions

EGF mapping offers a novel framework for possibly classifying and treating patients with AF based on the underlying pathophysiology of their AF disease rather than an estimation of the temporal duration of their AF episodes. In addition to source activity, EGF parameters quantifying fractionation and flow consistency are under development. It is well established that sources of excitation in AF may exist in areas with substrate abnormalities as evidenced by fractionated electrical potentials. Similarly, the EGF pattern can determine whether areas of conduction are stable in flow direction over time or whether they show a high flow angle variability (FAV), which measures how many degrees the flow vector angle changes on average from frame to frame. In a retrospective analysis by Spitzer et al., three EGF parameters: source activity, FAV and fractionation were determined in basket recordings collected in the RA and LA from 199 patients originally treated with FIRM-guided ablation in four centers in Europe [45]. For each patient, the final most relevant recording was selected from all basket catheter recordings after all ablation was completed and was classified by the presence or absence of EGF mechanisms, which were then correlated with clinical outcomes at 12-month post-ablation and showed that those patients with no extra-PV mechanisms had better outcomes compared with those patients whose AF was driven by stable extra-PV mechanisms. The ability to mechanistically differentiate functional sources of AF could enable electrophysiologists to tailor their ablation strategy to each individual patient. The ability to predict clinical outcomes based on the dynamic alterations of these functional sources during the course of a procedure not only provides the operator actionable targets during the procedure but may also inform them when additional ablation will not further improve the patient’s outcome. More EGM and outcomes data are needed and a prospective, randomized, controlled trial, *FLOW-AF* (NCT 04,473,963), is currently being completed to collect additional data on EGF source phenotypes, the ability to identify and acutely eliminate relevant sources and potentially improve clinical outcomes across a population of persistent and long-standing persistent AF patients enrolled at multiple centers.

## Clear unmet need for AF mapping

For persistent AF patients with recurrence despite intact PVI, ablation strategies remain poorly defined. The need for advanced mapping to identify extra-PV sources of AF will likely become increasingly evident as our ability to perform durable PVI improves. This ability to dynamically detect active sources of AF outside the PVs enables the classification of patients based on functional AF mechanisms—at the simplest level, the presence or absence of these EGF-identified sources offers a framework for distinguishing those patients with extra-PV sources of AF versus those with PV-triggered AF. Phenotyping patients based on their underlying AF pathophysiology rather than just the temporal persistence of their documented AF episodes may improve our ability to individualize AF ablation strategies, e.g., patients with PV-triggered AF only require PVI, while patients with extra-PV drivers need adjunctive ablation beyond the PVs.

## Conclusions

EGF mapping is an innovative AF mapping method based on well-established principles of optical flow and fluid dynamics. EGF mapping offers the ability to generate complete, near real-time temporospatial visualizations of atrial electrical wavefront propagation quickly and efficiently for the identification of putative AF sources. Although the electrical flow fields in AF are characteristically highly variable, the origins of EGF—defined as sources—frequently appear spatially conserved. Summary EGF maps aggregate and display the dominant patterns of excitation wave propagation from each of the 2 s flow vector maps and by integrating this repetitive behavior of source activity over time, provide a visual organization of the otherwise chaotic AF flow fields.
